# A dynamic nomogram for predicting the probability of irreversible neurological dysfunction after cervical spinal cord injury: research based on clinical features and MRI data

**DOI:** 10.1186/s12891-023-06570-z

**Published:** 2023-06-05

**Authors:** Si Chen, Guangzhou Li, Feng Li, Gaoju Wang, Qing Wang

**Affiliations:** 1 Department of Orthopaedics, People’s Hospital of Chongqing Banan District, Chongqing, China; 2 Department of Orthopaedics, The Affiliated Hospital of Southwest Medical University, No. 25 Taiping Street, Jiangyang District, Sichuan, 646000 China; 3 Department of Orthopaedics, The Affiliated Chengdu 363 Hospital of Southwest Medical University, Chengdu, China

**Keywords:** Cervical spinal cord injury, Neurological dysfunction, MRI, Dynamic nomogram, Outcome

## Abstract

**Background:**

Irreversible neurological dysfunction (IND) is an adverse event after cervical spinal cord injury (CSCI). However, there is still a shortage of objective criteria for the early prediction of neurological function. We aimed to screen independent predictors of IND and use these findings to construct a nomogram that could predict the development of neurological function in CSCI patients.

**Methods:**

Patients with CSCI attending the Affiliated Hospital of Southwest Medical University between January 2014 and March 2021 were included in this study. We divided the patients into two groups: reversible neurological dysfunction (RND) and IND. The independent predictors of IND in CSCI patients were screened using the regularization technique to construct a nomogram, which was finally converted into an online calculator. Concordance index (C-index), calibration curves analysis and decision curve analysis (DCA) evaluated the model's discrimination, calibration, and clinical applicability. We tested the nomogram in an external validation cohort and performed internal validation using the bootstrap method.

**Results:**

We enrolled 193 individuals with CSCI in this study, including IND (*n *= 75) and RND (*n* = 118). Six features, including age, American spinal injury association Impairment Scale (AIS) grade, signal of spinal cord (SC), maximum canal compromise (MCC), intramedullary lesion length (IMLL), and specialized institution-based rehabilitation (SIBR), were included in the model. The C-index of 0.882 from the training set and its externally validated value of 0.827 demonstrated the model's prediction accuracy. Meanwhile, the model has satisfactory actual consistency and clinical applicability, verified in the calibration curve and DCA.

**Conclusion:**

We constructed a prediction model based on six clinical and MRI features that can be used to assess the probability of developing IND in patients with CSCI.

**Supplementary Information:**

The online version contains supplementary material available at 10.1186/s12891-023-06570-z.

## Introduction

Affected by factors such as economic development, the incidence of traumatic spinal cord injury has continued to rise in recent years, which has attracted increasing attention from orthopedic surgeons [1, 2]. In 2019, there were 0.9 million incident cases of SCI worldwide, with falls and road injuries leading causes [3]. Spinal cord injury (SCI) is a severe pathological state because of its characteristics of high disability and mortality, seriously harming human health and the social economy [4, 5]. In developed countries, the total direct cost of care for patients with SCI exceeds billions of dollars annually, with an average lifetime cost of between $ 500,000 and $ 2 million for a patient with SCI [6, 7]. Although modern medicine is sufficiently advanced, effective treatment options for these patients are still lacking, and their prognosis is also challenging to grasp [8].

More than half of the injuries occurred at the cervical level [9]. CSCI tends to affect a broader range of limb dysfunction than other segments. Injury in this region can lead to neurological dysfunction such as flaccid paralysis, central cord syndrome, and Brown-Sequard syndrome. Whether neurological dysfunction can be improved has been the most concerning topic. Early understanding of the prognosis of patients is of great significance in better comprehending the severity of the injury and formulating individualized treatment plans. However, neurological recovery often takes a long time to reach the plateau, with some patients even lasting for one to two years [10, 11]. Therefore, it is hard to accurately evaluate the neurological outcome in the early stage of injury.

The American Spinal Injury Association Impairment Scale (AIS) has been revised several times since it was proposed, and it has become an international standard for evaluating the degree of SCI [12]. Many studies have confirmed the correlation between AIS grade and the outcome of neurological function, and the AIS grade is often used to determine the prognosis roughly [13–15]. However, this evaluation index is independent of clinical characteristics, imaging data, and treatment intervention and has significant limitations. In this study, we attempted to integrate multiple factors to develop a model to evaluate the neurological prognosis of CSCI patients more accurately. We hope to provide new ideas for the clinical treatment and risk identification of neurological dysfunction after CSCI.

## Patients and methods

### Patient selection

The data of patients with acute CSCI hospitalized in the Affiliated Hospital of Southwest Medical University from January 2015 to March 2021 were retrospectively analyzed as a training cohort. Subsequently, the data of patients with acute CSCI hospitalized in the Affiliated Chengdu 363 Hospital of Southwest Medical University during the same period externally validated our results.

The inclusion criteria are as follows: 1) Patients had a history of trauma and were diagnosed with CSCI; 2) The imaging data was complete and consistent with symptoms and signs; 3) Spinal shock resolved. The patients below are not qualified for this study: 1) Patients with previous neurological paralysis symptoms before this trauma; 2) Patients with severe craniocerebral trauma; 3) Patients with spinal cord malformation such as syringomyelia, basilar invagination, hemivertebra deformity, split cord malformation, etc.; 4) The history before admission more than one month; 5) Patients who did not receive surgical treatment; 6) Incomplete data; 7) Patients of less than 14 years of age. The Medical Ethics Committee of the Affiliated Hospital of Southwest Medical University approved this study.

### Observation indicators

The following data were documented for the study population: Age, gender, etiology, AIS grade [16], calcium, potassium, white blood cells (WBC), neutrophil to lymphocyte ratio (NLR), mean arterial pressure (MAP), signal of spinal cord (SC), maximum canal compromise (MCC), intramedullary lesion length (IMLL), operation timing, surgery duration, surgical approach, specialized institution-based rehabilitation (SIBR).

**Fig. 1:**
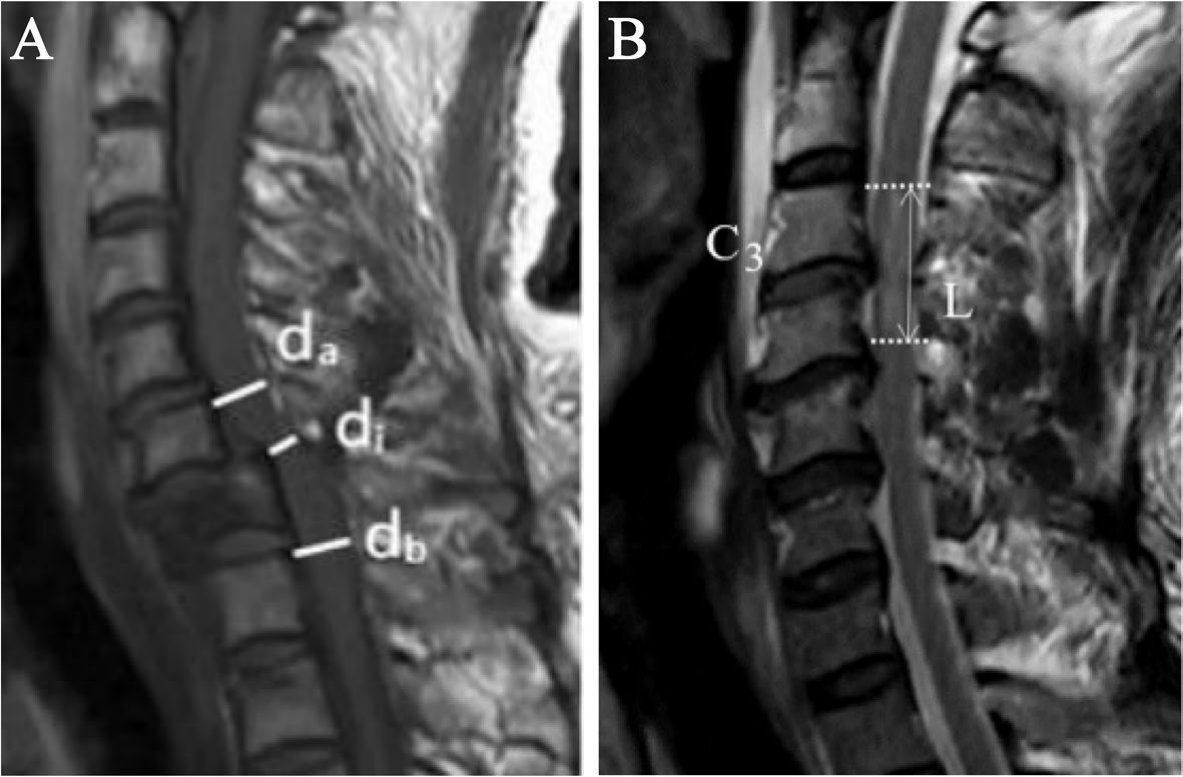
Measurement methods of MCC and IMLL. Abbreviations: MCC: maximum canal compromise; IMLL: intramedullary lesion length. Notes: (**A**) Select the median sagittal image of the magnetic resonance imaging, d_i_ represents the anteroposterior canal diameter of the maximum damage level, while d_a_ and d_b_ represent the diameter of anteroposterior canal diameter nearest to the normal level above and below the injury site, respectively. MCC (%) = 1-[ 2d_i_ / (d_a_ + d_b_)] × 100%. **B** Select the median sagittal image of the magnetic resonance imaging, and the rostrocaudal length of damage was measured in centimeters. The normal intramedullary signal was recorded as zero

Detailed clinical information was acquired from the medical record. All laboratory indicators and MAP were obtained from data collected within 24 h of admission. We divided the etiology into traffic-accident, high falls, struck by falling, low falls, and other traumatic causes. The signal of SC was classified as normal and abnormal based on MRI findings, with abnormalities including spinal cord edema, hemorrhage, and transection. On the basis of T1-MRI results [17], the MCC was calculated by measuring changes in the anteroposterior diameter of the spinal canal at the maximum level of injury and was further divided into three grades (< 30%, 30%-50%, > 50%) (Fig. 1A). IMLL was defined as the rostrocaudal length of high-signal intensity from the injury epicenter [18], measured twice times on MRT2WI, using average data (Fig. 1B). Operation timing was converted into binary variables early (< 72 h after injury) and late (≥ 72 h after injury). The surgical approach was divided into the anterior, posterior, and posterior-anterior combined approaches. The definition of SIBR was receiving treatment under the guidance of specialized rehabilitation institutions.

In order to make the prediction more accurate, we analyzed two important factors that may affect the efficacy of SIBR, including the start time of SIBR and the length of SIBR time.

Laboratory indices were assayed using a Sysmex XE-2100 automated hematology analyzer and Siemens Advia 2400 chemistry analyzer. Imaging data were scanned with Philips Intera Achieva 1.5 T MR machine. All images are uploaded to the iMedPacs system via a synchronous workstation and measured using the system's measurement tools.

### Outcome assessment

The outcome indicator of this study was the dichotomous variable of irreversible neurological dysfunction (IND) or reversible neurological dysfunction (RND) in CSCI patients. All patients were classified into IND and RND according to the following criteria: the differences in AIS grade at the baseline status and the observation time point from the twelve months. The improvement of AIS grade by at least one level was taken as the criterion for CSCI reversible neurological dysfunction. Telephone follow-up and outpatient follow-up medical records were used.

### Statistical analysis

R Studio ver. 4.1.2 and SPSS Statistics ver. 26.0 were used for statistical analysis. Continuous data were described as the means ± standard deviations or percentiles, while the categorical data were expressed in percentages. The *T*-test, Mann–Whitney U-test, and the chi-square test were used to retrospectively analyze the patients' differences in demographic and clinical characteristics. Statistical significance was confirmed at a two-tailed* P*-value lower than 0.05. The model was built, evaluated, and validated through R Studio.

Independent predictors were confirmed using the least absolute shrinkage and selection operator (LASSO) method. Visualization of the model was made possible with a static nomogram, which was eventually transformed into an online calculator to promote usage efficiency. The concordance index (C-index) estimated the model's discrimination, which could be replaced with the area under the receiver operating characteristic (ROC) curve (AUC). We also exploited the calibration curve analysis and the decision curve analysis (DCA) for model assessment. The calibration curve was performed to evaluate whether the predicted IND probability corresponded with the actual proportion. DCA was adopted to extrapolate the model's clinical applicability by calculating the net benefits at different threshold probabilities. Afterward, external validation of the model was conducted in the validation cohort, and it was also internally validated using the bootstrap method.

## Results

### Baseline characteristics

**Fig. 2:**
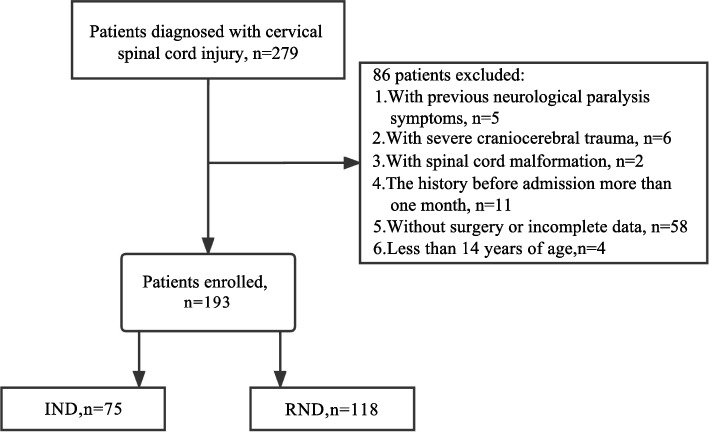
Patient flowchart. Abbreviations: IND: irreversible neurological dysfunction; RND: reversible neurological dysfunction

Data of 279 patients with CSCI were reviewed, and 193 patients were included in this study after excluding non-compliant patients, including 75 IND and 118 RND (Fig. 2). The demographic data and clinical features of patients were recorded in Table 1. There were 151 males (78.2%) and 42 females (21.8%). The median age was 54 years (42.5–60 years). The results showed that there were statistically significant differences between IND and RND in AIS grade, signal of SC, MCC, IMLL, and NLR (*P* **< **0.05). The baseline characteristics of the validation cohort were recorded in Table 2. There were no statistically significant differences in Age, AIS grade, signal of SC, MCC, IMLL and SIBR between the data from the training and validation cohorts.

**Table 1:** Baseline characteristics of the study population

**Variables**	**Overall (** ***n*** ** = 193)**	**IND (** ***n*** ** = 75)**	**RND (** ***n*** ** = 118)**	***P*** **-value**
**Age (years)**				0.151
**< 50**	82 (42.5%)	28 (37.3%)	54 (45.8%)	
**50–65**	75 (38.9%)	28 (37.3%)	47 (39.8%)	
** > 65**	36 (18.6%)	19 (25.4%)	17 (16.4%)	
**Gender, male/female**	151/42 (78.2/21.8%)	61/14 (81.3/18.7%)	90/28 (76.3/23.7%)	0.406
**Etiology**				0.190
**Traffic-accident**	39 (20.2%)	13 (17.3%)	26 (22.0%)	
**High falls**	80 (41.5%)	36 (48.0%)	44 (37.3%)	
**Struck by falling**	21 (10.9%)	8 (10.7%)	13 (11.0%)	
**Low falls**	51 (26.4%)	16 (21.3%)	35 (29.7%)	
**Others**	2 (1.0%)	2 (2.7%)	0 (0.0%)	
**AIS (grade)**				< 0.001
**A**	31 (16.1%)	27 (26.0%)	4 (3.4%)	
**B**	21 (10.9%)	8 (10.7%)	13 (11.0%)	
**C**	48 (24.9%)	6 (8.0%)	42 (35.6%)	
**D**	93 (48.1%)	34 (45.3%)	59 (50.0%)	
**MAP**	94.0 (87.0, 106.0)	95.0 (87.0, 108.0)	93.0 (86.0, 105.0)	0.191
**Calcium**	2.3 ± 0.2	2.3 ± 0.2	2.2 ± 0.1	0.377
**Potassium**	4.1 ± 0.4	4.1 ± 0.4	4.1 ± 0.5	0.745
**WBC**	9.0 (7.2, 11.5)	9.0 (7.6, 11.6)	8.9 (6.9, 11.5)	0.444
**NLR**	7.7 (3.5, 12.3)	9.3 (3.5, 13.2)	5.5 (3.4, 11.4)	0.037
**Signal of SC**				< 0.001
**Normal**	56 (29.0%)	7 (9.3%)	49 (41.5%)	
**Abnormal**	137 (71.0%)	68 (90.7%)	69 (58.5%)	
**MCC (%)**				< 0.001
**< 30**	94 (48.7%)	25 (33.3%)	69 (58.5%)	
**30–50**	53 (27.5%)	18 (24.0%)	35 (29.7%)	
**> 50**	46 (23.8%)	32 (42.7%)	14 (11.8%)	
**IMLL (cm)**	1.9 (0.0, 3.8)	3.8 (1.8, 5.1)	0.9 (0.0, 2.3)	< 0.001
**Operation timing**				0.454
**< 72 h**	24 (12.4%)	11 (14.7%)	13 (11.0%)	
**≥ 72 h**	169 (87.6%)	64 (85.3%)	105 (89.0%)	
**Surgery duration (min)**	120.0 (95.0, 167.5)	125.0 (90.0, 200.0)	120.0 (98.8, 160.0)	0.732
**Surgical approach**				0.546
**Anterior**	121 (62.7%)	46 (61.3%)	75 (63.6%)	
**Posterior**	61 (31.6%)	23 (30.7%)	38 (32.2%)	
**Posterior-anterior**	11 (5.7%)	6 (8.0%)	5 (4.2%)	
**SIBR, yes/no**	28/165 (14.5/85.5%)	7/68 (9.3/90.7%)	21/97 (17.8/82.2%)	0.104

*Abbreviations*: *AIS* American Spinal Injury Association Impairment Scale, *MAP* mean arterial pressure, *WBC* white blood cells, *NLR* neutrophil to lymphocyte ratio, *SC* spinal cord, *MCC* maximum canal compromise, *IMLL* intramedullary lesion length, *SIBR* specialized institution-based rehabilitation, *IND* irreversible neurological dysfunction, *RND* reversible neurological dysfunction

**Table 2:** Comparison of the variable characteristics of the validation and training cohort

**Variables**	**Overall**	**Baseline characteristics comparison**		***P***	**Baseline characteristics comparison**		***P***
		**IND (** ***n*** ** = 30)**	**RND (** ***n*** ** = 50)**		**training cohort (** ***n*** ** = 193)**	**validation cohort (** ***n*** ** = 80)**	
**Age (years)**				0.083			0.268
**< 50**	34 (42.5%)	8 (26.7%)	26 (52.0%)		82 (42.5%)	34 (42.5%)	
**50–65**	37 (46.3%)	18 (60.0%)	19 (38.0%)		75 (38.9%)	37 (46.3%)	
**> 65**	9 (11.2%)	4 (13.3%)	5 (10.0%)		36 (18.6%)	9 (11.2%)	
**AIS (grade)**				0.011			0.146
**A**	13 (16.3%)	10 (33.3%)	3 (6.0%)		31 (16.1%)	13 (16.3%)	
**B**	14 (17.5%)	4 (13.3%)	10 (20.0%)		21 (10.9%)	14 (17.5%)	
**C**	11 (13.7%)	2 (6.7%)	9 (18.0%)		48 (24.8%)	11 (13.7%)	
**D**	42 (52.5%)	14 (46.7%)	28 (56.0%)		93 (48.2%)	42 (52.5%)	
**Signal of SC**				0.005			0.568
**Normal**	26 (32.5%)	4 (13.3%)	22 (44.0%)		56 (29.0%)	26 (32.5%)	
** Abnormal**	54 (67.5%)	26 (86.7%)	28 (56.0%)		137 (71.0%)	54 (67.5%)	
**MCC (%)**				< 0.001			0.278
** < 30**	37 (46.3%)	9 (30.0%)	28 (56.0%)		94 (48.7%)	37 (46.3%)	
**30–50**	29 (36.2%)	9 (30.0%)	20 (40.0%)		53 (27.5%)	29 (36.2%)	
**> 50**	14 (17.5%)	12 (40.0%)	2 (4.0%)		46 (23.8%)	14 (17.5%)	
**IMLL (cm)**	1.7 (0.0, 3.7)	3.6 (1.2, 4.8)	1.0 (0.0, 2.7)	< 0.001	1.9 (0.0, 3.8)	1.7 (0.0, 3.7)	0.680
**SIBR, yes/no**	16/64 (20.0/80.0%)	7/23 (23.3/76.7%)	9/41 (18.0/82.0%)	0.564	28/165 (14.5/85.5%)	16/64 (20.0/80.0%)	0.261

*Abbreviations*: *AIS* American Spinal Injury Association Impairment Scale, *SC* spinal cord, *MCC* maximum canal compromise, *IMLL* intramedullary lesion length, *SIBR* specialized institution-based rehabilitation, *IND* irreversible neurological dysfunction, *RND* reversible neurological dysfunction

In addition, we obtained detailed data on 38 patients who received SIBR. As shown in Table S1, the length of SIBR time and the start time of SIBR were not significantly different in the IND and RND groups. Pairwise comparison within the group found that receiving SIBR within one month after injury had a higher recovery rate than receiving SIBR more than three months after injury ( *P* = 0.032). After Bonferroni correction ( *P'=*0.05 / [ k ( k-1) / 2] = 0.017, *P'* **<** *P*), the difference between these two groups was not considered to be statistically significant.

### Model construction

**Fig. 3:**
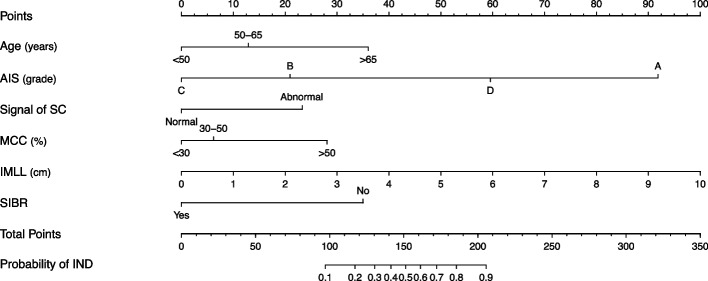
Nomogram for predicting IND after CSCI in the training cohort. Abbreviations: IND: irreversible neurological dysfunction; CSCI: cervical spinal cord injury; AIS: American spinal injury association Impairment Scale; SC: spinal cord; MCC: maximum canal compromise; IMLL: intramedullary lesion length; SIBR: specialized institution-based rehabilitation. Notes: The nomogram shows the six independent predictors of the model, with the abscissa being a graphical score assigned to the predictor. The sum of these six scores generated a plot on the “Total points” axis, with the corresponding values below representing the probability of IND after CSCI

After the LASSO variables selection, we screened six independent predictors, including age, AIS grade, signal of SC, MCC, IMLL, and SIBR (Figure S1). Based on these indicators, a model by multivariate logistic regression was developed and presented in the form of a static nomogram (Fig. 3). Their odds ratio (OR) values and regression coefficients are displayed in Table 3. In addition, we simplified the model to a web calculator whose access link is https://CSCI.shinyapps.io/dynnomapp/ (Figure S2).

**Table 3:** Multivariate logistic regression model in the training cohort

**Variables**	**Regression coefficient**	**OR (95%CI)**	***P***
**Age (year)**			
**50–65**	0.522	1.685 (0.682 − 4.277)	0.262
**> 65**	1.460	4.305 (1.388 − 14.211)	0.013
**AIS (grade)**			
**B**	0.849	2.336 (0.537 − 10.537)	0.258
**D**	2.413	11.171 (3.591–43.278)	< 0.001
**A**	3.722	41.325(8.141–293.000)	< 0.001
**Signal of SC = “abnormal”**	0.944	2.571(0.769–9.029)	0.129
**MCC (%)**			
**30–50**	0.251	1.285(0.486–3.345)	0.608
**> 50**	1.138	3.120(1.111–9.135)	0.033
**IMLL**	0.405	1.500(1.119–2.068)	0.009
**SIBR = “No”**	1.419	4.131(1.057–21.978)	0.061

*Abbreviations*: *AIS* American Spinal Injury Association Impairment Scale, *SC* spinal cord, *MCC* maximum canal compromise, *IMLL* intramedullary lesion length, *SIBR* specialized institution-based rehabilitation, *OR* odds ratio, *CI* confidence interval

### Model evaluation

**Fig. 4:**
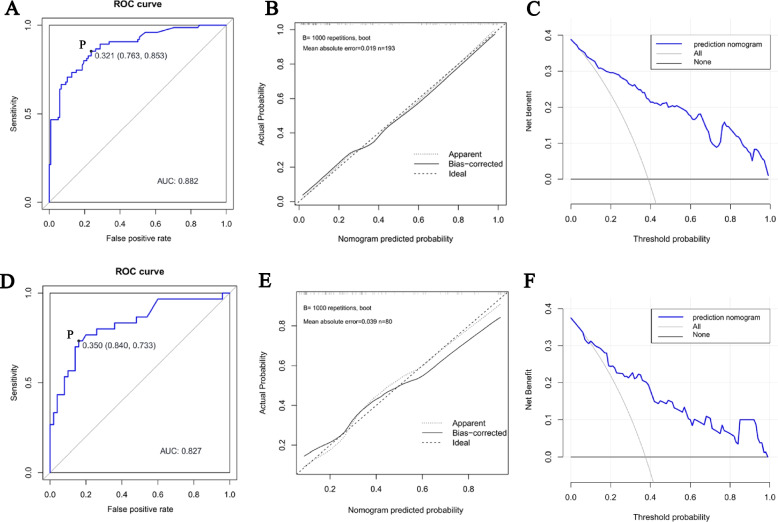
Validation of nomogram. Abbreviations: ROC: receiver operating characteristic; AUC: area under the receiver operating characteristic. Notes: Plots **A**, **B**, and **C** are from the training cohort, and plots **D**, **E**, and **F** are from the validation cohort. Receiver operating characteristic curve of the nomogram (**A** and **D**). Point P is the threshold probability when the model has optimal sensitivity and specificity. Calibration curves of the nomogram (**B** and **E**). The ideal line indicates that the predicted probability perfectly coincides with the actual occurrence of IND, the apparent line reflects the prediction performance of the established model, and the distance between these two lines reflects the actual consistency of the model. Decision curve analysis of the nomogram (**C** and **F**). The X-axis represents the threshold probability, and the Y-axis represents the net benefit. The model has better clinical applicability when the net benefit of the nomogram is greater than the All and None lines at the same threshold

The C-index of 0.882 (95%CI: 0.833 – 0.931) in the training cohort demonstrated a good discriminant ability. The threshold possibility was 0.321, with the optimal sensitivity of 0.763 and specificity of 0.853 (Fig. 4A). In the calibration curve, the predicted curve and the reference curve (45-degree diagonal) roughly overlap, suggesting that the predicted incidence of outcome events was highly consistent with the actual situation (Fig. 4B). DCA shows the model was clinically beneficial (Fig. 4C). The C-index of 0.891 from the internal bootstrap validation verified the stability of the model. In the validation cohort, the C-index was 0.827 (95%CI: 0.778 – 0.876) (Fig. 4D), which corresponded with our results. Meanwhile, the calibration curve (Fig. 4E) and DCA (Fig. 4F) showed that the model had satisfactory actual consistency and clinical applicability.

## Discussion

Acute CSCI is a severe nerve trauma, and its prognosis varies greatly. Several scholars have confirmed that the prognosis of SCI is related to many factors, such as injury specificity and post-injury management [10, 19]. However, the weight of each element on the prognosis is different. Therefore, it is unreasonable to use only one feature as a decisive factor for estimating the outcome of patients with SCI. The nomogram solves these problems well and assigns different scores to each prediction factor to visually present the probability of outcome events.

A total of 16 clinical and imaging features were analyzed in our study. After Lasso regression, the six indicators were included: age, AIS grade, signal of SC, MCC, IMLL, and SIBR. Age, AIS grade, signal of SC, MCC, and IMLL could be easily obtained directly during hospitalization. SIBR is indirectly achieved by understanding the wishes of patients. Therefore, the nomogram developed in this study can be used as an easy-to-use tool for clinical application. We confirmed the model's high accuracy, stability, and clinical applicability. In addition, we created an online calculator for clinical application. Doctors could conveniently obtain the possibility of IND in patients with CSCI through a mobile terminal.

Previous studies have suggested that age may not be related to motor recovery. However, the results obtained without variable stratification are questionable, given the heterogeneity of clinical characteristics and treatment options in CSCI patients. Furlan et al. [20] found that the potential of older patients with SCI to improve within the first year post-injury neurologically does not appear to translate into similar functional recovery compared to that seen in younger individuals (< 65 years). Some scholars also reported that compared with patients under 50 years old, the elderly were less likely to achieve walking function post-injury [21]. Furthermore, we conducted a more detailed grouping of age, and we still found that age can be screened as an independent predictor of IND after CSCI. Overall, older age appears to be associated with poorer neurological recovery, although the exact age is unclear [11]. These results may be related to reduced antioxidant defenses and axonal plasticity in elderly patients [22, 23]. In addition, negative management attitudes due to ageism and multimorbidity play a non-negligible role [24, 25].

In many studies, the severity of SCI is considered the most critical factor in predicting neurological outcomes. As a recognized standard for evaluating neurological function in SCI, AIS shows good overall discrimination in predicting the outcome of neurological function in patients. In the model established in this study, both AIS A (OR = 41.325) and AIS D (OR = 11.171) had a significant odds ratio, indicating a strong correlation between AIS as a predictor and outcome events. Previous literature has shown that the recovery rate of neurological function in SCI patients met the rule of grade C of AIS > B > D > A [10, 26, 27], which is consistent with the score of each grade in this model. Interestingly, although grade D of AIS is the lightest in injury classification, its neurological prognosis is not the best. The ceiling effect of the AIS grade may have contributed to this result.

As an essential examination for patients with CSCI, MRI reflects the state of spinal cord after injury and the degree of violence [28]. Boese et al. [29] found that the signal of SC was associated with AIS grade improvement in adult patients with SCI without radiologic abnormalities. They believed that patients with normal MRI findings had a better prognosis than those with abnormalities, while patients with extraneural abnormalities (compression) had a better prognosis than patients with intraneural abnormalities (edema, hemorrhage, contusion, and transection). Furthermore, Fehlings et al. [30] reported that SCI patients with the signal of SC changes showed worse neurological function during one follow-up. Our results coincided with the previously proposed views. We also find that the probability of IND increases with the increase of IMLL. Some scholars reported IMLL increased by 10 mm, and the AIS grade conversion rate can be reduced by 40% [31]. Matsushita et al. [32] concluded that patients were more likely to achieve walking function at discharge when the vertical diameter of the T2 high-intensity area was less than 45 mm. Another study found that incomplete SCI with lesion length greater than 36 mm was independently associated with a more severe AIS grade one year later [33]. It is worth noting that the IMLL does not always remain constant, which also gives the model the potential for dynamicity. In addition, World Federation of Neurosurgical Societies Spine Committee members and experts agree that MCC is an important prognostic indicator [34]. More significant spinal cord compression often leads to more severe neurological dysfunction [35], while MCC shows the extent of spinal cord compression in the plane of injury. In this study, patients with MCC less than 30% had the highest recovery rate at follow-up, while those with MCC greater than 50% had the worst prognosis, which is consistent with the above view. The cause of this result may be related to persistent compression of the spinal cord triggered by serious canal compromise. Furthermore, we believe that the spinal cord suffered greater instantaneous violence at serious canal compromise.

Although the contribution of rehabilitation therapy to functional improvement remains controversial, the recent clinical practice guidelines still recommend rehabilitation treatment for patients with acute SCI who are stable and able to tolerate the required rehabilitation intensity [36]. Standardized rehabilitation reduces complications and positively affects neurological recovery, even in patients with AIS A [37]. The efficacy and application potential of some rehabilitation measures, such as hyperbaric oxygen therapy, electrical stimulation and hydrotherapy, has been confirmed in previous studies [38–40]. In general, low-intensity rehabilitation can begin within three days of the injury, while high-intensity activities need to wait until at least four days after the injury to be relatively safe [41]. Earlier rehabilitation maximizes the important window of the first three months and offers greater potential for prognostic improvement. Herzer et al. [42] found that the prolonged time between injury and rehabilitation was associated with lower motor scores after one year. In addition, within a certain period, the length of rehabilitation time was also positively correlated with the final motor function score [37]. Our findings suggest a potential correlation between early rehabilitation interventions and better prognosis. Unlike previous studies, we found that the length of rehabilitation was not associated with prognosis, which may be attributed to injury specificity, population differences, and sample size. Although our study confirmed that patients receiving SIBR had a higher recovery rate, there was still a lack of a standardized strategy to guide rehabilitation treatment.

Several limitations of this study should be explained. First, this is a retrospective study that may have inherent information bias. Second, although the model has shown its superiority after verification by data from another center, the overall sample size of this study is small, which may not strictly meet the principle of events per variable. Third, the limited sample size of the earlier surgical (< 24 h) group due to various conditions was not included in the evaluation, which was considered an independent protective factor in previous studies. Finally, most of our cases were surgically treated, so we excluded cases without surgical records to reduce data bias. This may result in the model not being well applied to patients without surgery.

## Conclusion

Our study constructed a nomogram and online calculator based on six clinical and MRI features that can be used to identify patients with CSCI who are at risk of IND. Early prediction of IND occurrence is expected to guide clinicians in providing personalized support and treatment strategies.

## Supplementary Information

**Additional file 1: Table S1.** Comparison of rehabilitation factors and prognosis of neurological function.

**Additional file 2: Figure S1.** The process of screening independent predictors. **Figure S2.** Dynamic nomogram for predicting IND after CSCI.

## Data Availability

The authors declare the data analyzed during the current study could be available from the corresponding author upon reasonable request.

## Abbreviations

IND: Irreversible neurological dysfunction
CSCI: Cervical spinal cord injury
RND: Reversible neurological dysfunction
SCI: Spinal cord injury
AIS: American Spinal Injury Association Impairment Scale
WBC: White blood cells
NLR: Neutrophil to lymphocyte ratio
MAP: Mean arterial pressure
SC: Spinal cord
MCC: Maximum canal compromise
IMLL: Intramedullary lesion length
SIBR: Specialized institution-based rehabilitation
LASSO: Least absolute shrinkage and selection operator
C-index: Concordance index
ROC curve: Receiver operating characteristic curve
AUC: Area under the receiver operating characteristic
DCA: Decision curve analysis
OR: Odds ratio
