# Supplementary material for: A dynamic nomogram for predicting the probability of irreversible neurological dysfunction after cervical spinal cord injury: research based on clinical features and MRI data
Source: BMC Musculoskelet Disord. 2023 Jun 5;24:459. doi: 10.1186/s12891-023-06570-z (PMC10240743; doi:10.1186/s12891-023-06570-z)
Supplement: Supplementary file 1 — Additional file 1: Table S1. Comparison of rehabilitation factors and prognosis of neurological function. [file 12891_2023_6570_MOESM1_ESM.pdf]

Table S1 Comparison of rehabilitation factors and prognosis of neurological function

Group	IND (n=12)	RND (n=26)	P	P_{A-B}	P_{A-C}	P_{B-C}
**Start time of SIBR***			0.090	0.526	0.032	0.119
A (<1 month)	3	13				
B (1-3 months)	4	10				
C (> 3 months)	5	3				
**Length of SIBR time**			0.623	0.652	0.330	0.466
A (<3 week)	4	6				
B (3-8 weeks)	7	15				
C (> 8 weeks)	1	5				

Abbreviations: SIBR, specialized institution-based rehabilitation; IND, irreversible neurological dysfunction; RND, reversible neurological dysfunction.

Notes: * The time from injury to start receiving SIBR.
