# Supplementary material for: A dynamic nomogram for predicting the probability of irreversible neurological dysfunction after cervical spinal cord injury: research based on clinical features and MRI data
Source: BMC Musculoskelet Disord. 2023 Jun 5;24:459. doi: 10.1186/s12891-023-06570-z (PMC10240743; doi:10.1186/s12891-023-06570-z)
Supplement: Supplementary file 2 — Additional file 2: Figure S1. The process of screening independent predictors. Figure S2. Dynamic nomogram for predicting IND after CSCI. [file 12891_2023_6570_MOESM2_ESM.docx]

Figure S1. The process of screening independent predictors.


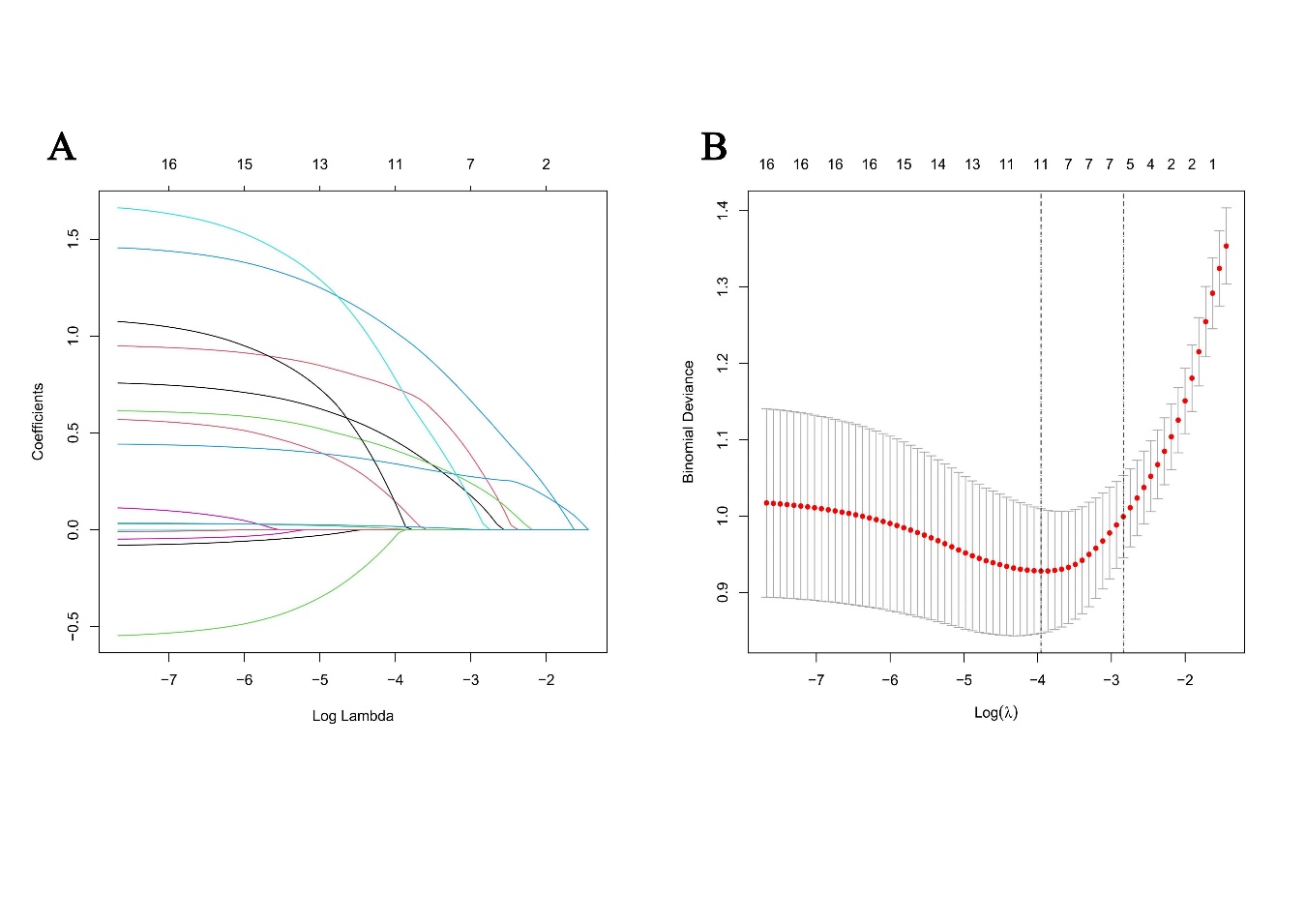


Abbreviations: LASSO, least absolute shrinkage and selection operator; SE, standard error.

Notes: (A) 16 variables to be analyzed are arrayed in the log (λ) sequence. Six predictors are chosen according to the 1-SE criteria in our study; (B) Prediction factors are screened by LASSO regression analysis. Based on the minimum criteria (left dotted line) and the 1-SE criteria (right dotted line), the optimal turning parameter (λ) is determined.

Figure S2. Dynamic nomogram for predicting IND after CSCI.


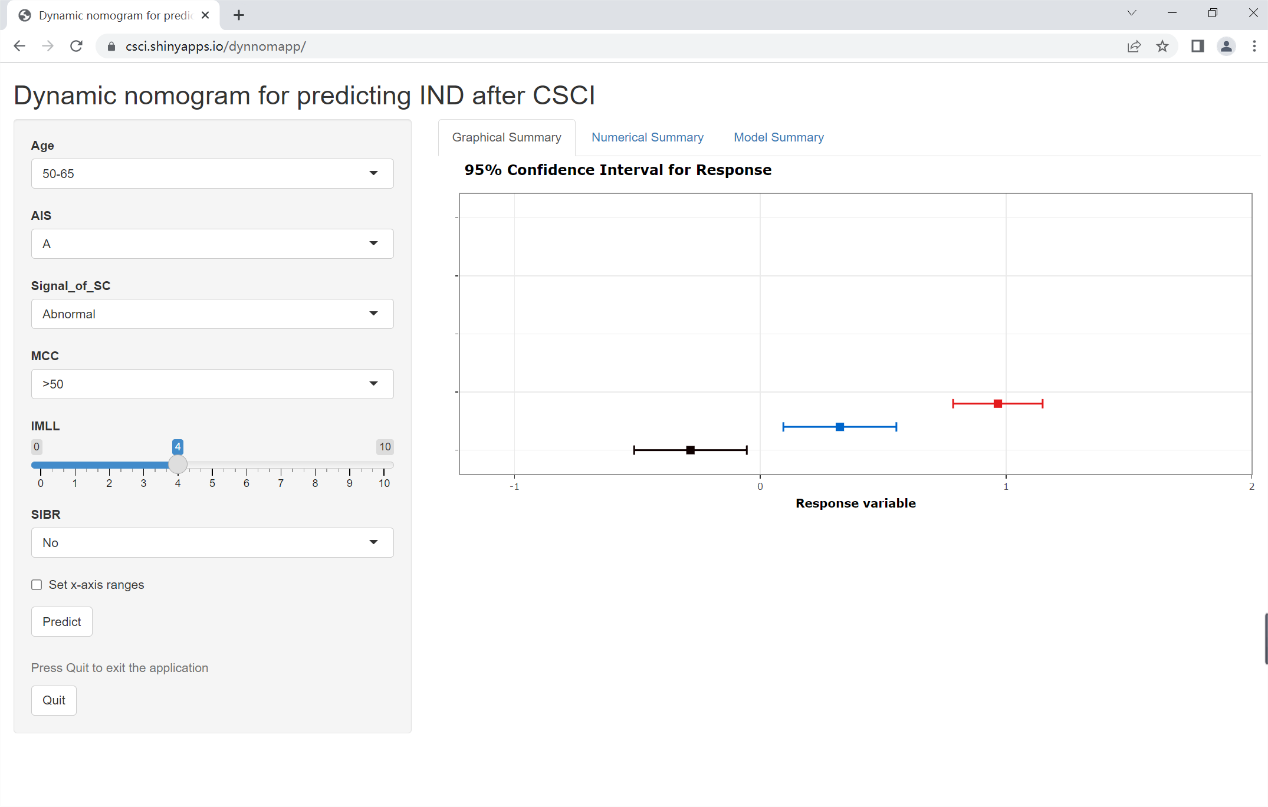


Abbreviations: IND, irreversible neurological dysfunction; CSCI, cervical spinal cord injury; AIS, American Spinal Injury Association Impairment Scale; SC, spinal cord; MCC, maximum canal compromise; IMLL, intramedullary lesion length; SIBR, specialized institution-based rehabilitation.

Notes: The results of the six independent predictors in the dynamic Web-based calculator can be selected, and their predictions are presented on the right side of the web page.
